# Screening of endogenous strong promoters for enhanced production of medium-chain-length polyhydroxyalkanoates in *Pseudomonas mendocina* NK-01

**DOI:** 10.1038/s41598-019-39321-z

**Published:** 2019-02-12

**Authors:** Fengjie Zhao, Xiangsheng Liu, Annie Kong, Yuxin Zhao, Xu Fan, Ting Ma, Weixia Gao, Shufang Wang, Chao Yang

**Affiliations:** 1 Key Laboratory of Molecular Microbiology and Technology for Ministry of Education, Nankai University, Tianjin, 300071 China; 2 State Key Laboratory of Medicinal Chemical Biology, Nankai University, Tianjin, 300071 China

## Abstract

Polyhydroxyalkanoate (PHA) can be produced by microorganisms from renewable resources and is regarded as a promising bioplastic to replace petroleum-based plastics. *Pseudomonas mendocina* NK-01 is a medium-chain-length PHA (mcl-PHA)-producing strain and its whole-genome sequence is currently available. The yield of mcl-PHA in *P. mendocina* NK-01 is expected to be improved by applying a promoter engineering strategy. However, a limited number of well-characterized promoters has greatly restricted the application of promoter engineering for increasing the yield of mcl-PHA in *P. mendocina* NK-01. In this work, 10 endogenous promoters from *P. mendocina* NK-01 were identified based on RNA-seq and promoter prediction results. Subsequently, 10 putative promoters were characterized for their strength through the expression of a reporter gene *gfp*. As a result, five strong promoters designated as P4, P6, P9, P16 and P25 were identified based on transcriptional level and GFP fluorescence intensity measurements. To evaluate whether the screened promoters can be used to enhance transcription of PHA synthase gene (*phaC*), the three promoters P4, P6 and P16 were separately integrated into upstream of the *phaC* operon in the genome of *P. mendocina* NK-01, resulting in the recombinant strains NKU-4C1, NKU-6C1 and NKU-16C1. As expected, the transcriptional levels of *phaC1* and *phaC2* in the recombinant strains were increased as shown by real-time quantitative RT-PCR. The *phaZ* gene encoding PHA depolymerase was further deleted to construct the recombinant strains NKU-∆*phaZ*-4C1, NKU-∆*phaZ*-6C1 and NKU-∆*phaZ*-16C1. The results from shake-flask fermentation indicated that the mcl-PHA titer of recombinant strain NKU-∆*phaZ*-16C1 was increased from 17 to 23 wt% compared with strain NKU-∆*phaZ*. This work provides a feasible method to discover strong promoters in *P. mendocina* NK-01 and highlights the potential of the screened endogenous strong promoters for metabolic engineering of *P. mendocina* NK-01 to increase the yield of mcl-PHA.

## Introduction

Currently, promoter engineering can serve as a powerful tool for rational tuning of the activity of the synthetic pathway enzymes for overproduction of many important bio-based chemicals^1–5^. Although various promoters may be obtained by construction and screening of promoter libraries^6–8^, library construction is a labor- and time-consuming task and screening of different promoters from libraries is inefficient. Transcriptome sequencing (RNA-seq) has provided an alternative strategy for the discovery of different types of endogenous promoters.

Microbial genomes are regarded as huge reservoirs for a variety of candidate endogenous promoters for metabolic pathway engineering. So far, a number of candidate endogenous promoters predicted by combination of RNA-seq and reporter gene assay have been applied for metabolic pathway optimization to improve the yield of target products. For example, 166 putative endogenous constitutive promoters from *Streptomyces coelicolor* M145 were predicted by RNA-seq, eight of which were further characterized by a reporter gene *gfp*, and four characterized promoters with different strengths were applied for the activation of cryptic biosynthetic clusters and resulted in different levels of the production of jadomycin B in *S. venezuelae* ISP5230^9^. In another study, 32 candidate endogenous promoters from *S. albus* J1074 were predicted by RNA-seq analysis, among which 10 strong promoters and four constitutive promoters were identified using a streptomycete reporter gene, *xylE*, and used for successful activation of a cryptic gene cluster from *S. griseus* in three widely used *Streptomyces* strains^10^. Song *et al*.^11^ identified a panel of stress-activated endogenous promoters by measuring the strengths of 84 predicted promoter sequences with a reporter gene *gfp* under specific stress conditions, and selected promoters elevated the final production of both cytoplasmic β-galactosidase and secreted protein α-amylase. In addition, six endogenous promoters from *Rhodotorula toruloides* were identified by luciferase reporter assay, among which three strong promoters were applied for overexpression of diacylglycerol acyltransferase for enhancing lipid accumulation in *R. toruloides*^12^. Yang *et al*.^13^ identified four classes of phase-dependent promoters with different strengths from 114 *Bacillus subtilis* endogenous promoters based on the database DBTBS and GFP reporter assay and the characterized phase-dependent promoters were applied for secretory expression of enzymes. In 2017, 104 native promoter-5′-UTR complexes (PUTR) which were screened from *Escherichia coli* based on a series of RNA-seq data were characterized by a reporter gene *gfp* and four engineered PUTRs showed stronger activities than the P_BAD_ promoter^14^.

Polyhydroxyalkanoates (PHA) are a family of biopolyesters synthesized by bacteria and archaea that accumulate as intracellular storage reserves of carbon and energy under the unbalanced growth conditions^15^. PHAs have attracted considerable attention as potential candidates to replace some oil-based plastics because of their biodegradability, biocompatibility, thermal and mechanical properties similar to plastics, and capability of being produced from renewable resources^16^. PHAs are traditionally classified into two major types, i.e., short-chain-length PHAs (scl-PHA) consisting of monomer repeat units of 3 to 5 carbon atoms and medium-chain-length PHAs (mcl-PHA) consisting of monomer repeat units of 6 to 14 carbon atoms.

Many members from the genus *Pseudomonas* have an ability to synthesize mcl-PHA via either fatty acid *de novo* biosynthesis pathway from unrelated carbon sources (e.g., glucose and glycerol) or β-oxidation pathway from related carbon sources (e.g., fatty acids)^17–20^. *Pseudomonas mendocina* NK-01, which was isolated by our lab from farmland soil, can synthesize mcl-PHA and alginate oligosaccharides (AO) simultaneously from glucose and the PHA synthase operon in this strain comprises two class II synthase genes *phaC1* and *phaC2* linked by a PHA depolymerase gene *phaZ*^21,22^. The mcl-PHA synthesized by *P. mendocina* NK-01 possesses superior physical properties and special monomer compositions^23^.

To date, whole-genome sequencing of *P. mendocina* NK-01 has been completed^24^ and a genome editing system has been developed for *P. mendocina* NK-01^25^, which have paved the way for metabolic pathway engineering of *P. mendocina* NK-01. In this work, five endogenous promoters from *P. mendocina* NK-01 were identified based on RNA-seq analysis, promoter prediction and GFP reporter assay, three of which were used to enhance transcription of *phaC* by integrating each promoter into the genome of *P. mendocina* NK-01. When combined with deletion of *phaZ*, the recombinant strain NKU-∆*phaZ*-16C1 had a 6% increase in mcl-PHA titer compared with NKU-∆*phaZ*.

## Results and Discussion

### Screening of endogenous strong promoters from *P. mendocina* NK-01 via RNA-seq analysis and promoter prediction

For RNA-seq analysis, transcriptional level of a gene is positively correlated with RPKM value^26^. Through RNA-seq analysis of *P. mendocina* NK-01, transcriptional levels of all genes were ranked from high to low based on their RPKM values. The first 30 genes ranked by RPKM values were assumed to be highly active at the transcriptional level (Table S1). Thus, the upstream regions of the 30 genes with high RPKM values were selected as the detection targets for promoter prediction. Through further screening using an online promoter prediction software, 10 out of 30 candidate sequences were identified as the putative promoter sequences (Fig. S1) and selected for subsequent cloning and characterization (Table 1).

**Table 1:** Selection of the 10 highly expressed genes in RNA-seq for promoter cloning.

Gene ID	Productions	Promoters	Length of promoters (bp)	RPKM
MDS_2118	hypothetical protein	P4	423	24593.43
MDS_0756	hypothetical protein	P6	261	24066.04
MDS_3450	hypothetical protein	P9	177	16182.31
MDS_2531	alcohol dehydrogenase	P16	199	8949.33
MDS_0957	ribosome-associated translation inhibitor RaiA	P17	85	8412.46
MDS_0563	poly(hydroxyalkanoate) granule-associated protein	P18	183	8222.26
MDS_1497	17 kDa surface antigen/outer membrane lipoprotein	P20	102	7267.13
MDS_1161	arginine/ornithine antiporter	P23	394	6935.83
MDS_4257	outer membrane protein W	P25	178	6637.68
MDS_3908	transport-associated protein	P29	419	5888.12

### Cloning of strong promoters from *P. mendocina* NK-01

The promoter regions of the 10 highly expressed genes were PCR-amplified from the genomic DNA of *P. mendocina* NK-01. To obtain the intact promoter sequence of each of the 10 highly expressed genes, in this work, the entire intergenic region between the highly expressed gene and its upstream gene was selected as the target region to be cloned by PCR, except for the native ribosomal binding site (RBS). The results from DNA sequencing showed that the cloned DNA fragments coincided with the selected intergenic regions at the nucleotide level (data not shown).

### Characterization of the cloned promoters via qPCR

**Figure 1:**
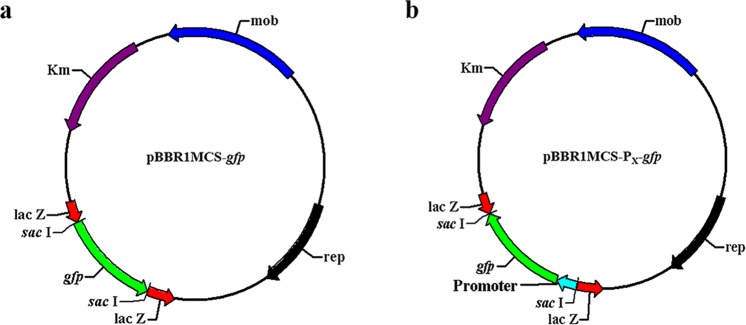
Recombinant plasmids for promoter characterization with *gfp* as a reporter gene. (**a**) Recombinant plasmid with a *lac* promoter as a control. (**b**) Recombinant plasmids for characterizing the strengths of 10 selected endogenous promoters.

**Figure 2:**
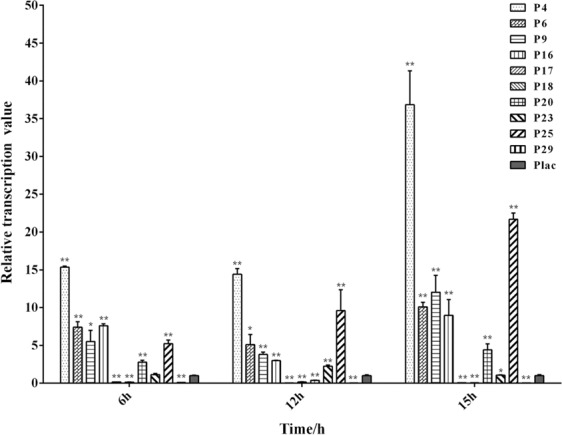
Characterization of the chosen promoters and *lac* promoter via qPCR analysis. Transcription of *gfp* gene under different promoters in *P. mendocina* NKU was quantified at different growth phases. 16S rDNA gene was used as internal reference. The relative transcription value of *gfp* gene under *lac* promoter was set as 1. Data represent the mean values ± standard deviations of triplicate measurements from three independent experiments. A Student’s *t*-test was performed between *lac* promoter and chosen promoters. * and ** indicate *P* < 0.05 and *P* < 0.01, respectively.

To assess the strengths of the cloned promoters, the promoters sequences were fused to the 5′-end of the amplified *gfp* gene and then inserted into a broad-host-range cloning vector pBBR1MCS-2 able to replicate in various gram-negative bacteria^27^ using homologous recombination (Fig. 1). qPCR was employed for the analysis of transcriptional levels of *gfp* under different promoters at different growth phases, i.e., early log-phase (6 h), post log-phase (12 h) and stationary phase (15 h) (Fig. S2). Among the 10 tested promoters, the transcriptional levels of the five promoters P4, P6, P9, P16 and P25 were much higher than that of *lac* promoter at different growth phases. Compared with *lac* promoter, the strongest promoter P4 showed a 36-fold increase in the transcriptional activity at the stationary phase (Fig. 2). When detecting with most of the cloned promoters, the transcriptional levels of reporter gene *gfp* varied significantly at different growth phases. The five strong promoters P4, P6, P9, P16 and P25 had higher transcriptional levels in post log-phase than stationary phase or early log-phase (Fig. S3). In contrast, relatively minor differences in transcriptional levels were detected with the five strong promoters between stationary phase and early log-phase (Fig. S3). The promoter P16 had a relatively stable transcriptional activity throughout the growth period (Fig. S3). In previous studies, different types of promoters including strong promoters, growth phase-dependent promoters and constitutive promoters have been well characterized^10,13^. Because of good system compatibility with the host cell, the selection of endogenous promoters may be more practical and purposeful for their applications in synthetic biology and metabolic engineering of the host itself.

The promoters P17, P18, and P29 had high RPKM values in the RNA-seq analysis, but the promoters placed on plasmid exhibited lower transcriptional levels than *lac* promoter at any growth phase (Fig. 2). The chosen endogenous promoters function well in the genome, which might be attributed to the assistance of the nearby regulatory sequences. Once the promoters were cloned separately, they did not work well.

Since the screened endogenous promoters were expected to be used for improving PHA production, the RNA-seq data were obtained with *P. mendocina* grown in PHA fermentation medium. For characterization of the cloned promoters by a reporter gene assay, the commonly used LB medium for the various reporter gene assays was also selected for this study^11,13^. The well-characterized promoters in LB medium may also have the potential to be applied for the synthesis of other products in *P. mendocina*. However, the transcriptional levels of the 10 candidate promoters obtained by RNA-seq analysis may not always be consistent with the transcriptional levels measured by qPCR due to the different culture conditions. For example, the promoters P17, P18 and P29 had high RPKM values in the RNA-seq analysis, but they exhibited lower transcriptional levels in the reporter gene assay than *lac* promoter at any growth phase. In the future, more RNA-seq data based on different culture conditions should be overall considered to select the candidate promoters. Then, through characterization of the putative promoters by a reporter gene assay in LB medium, the screened strong endogenous promoters may have a wide-range application for various products in *P. mendocina*.

### Characterization of the cloned promoters via GFP fluorescence measurement

**Figure 3:**
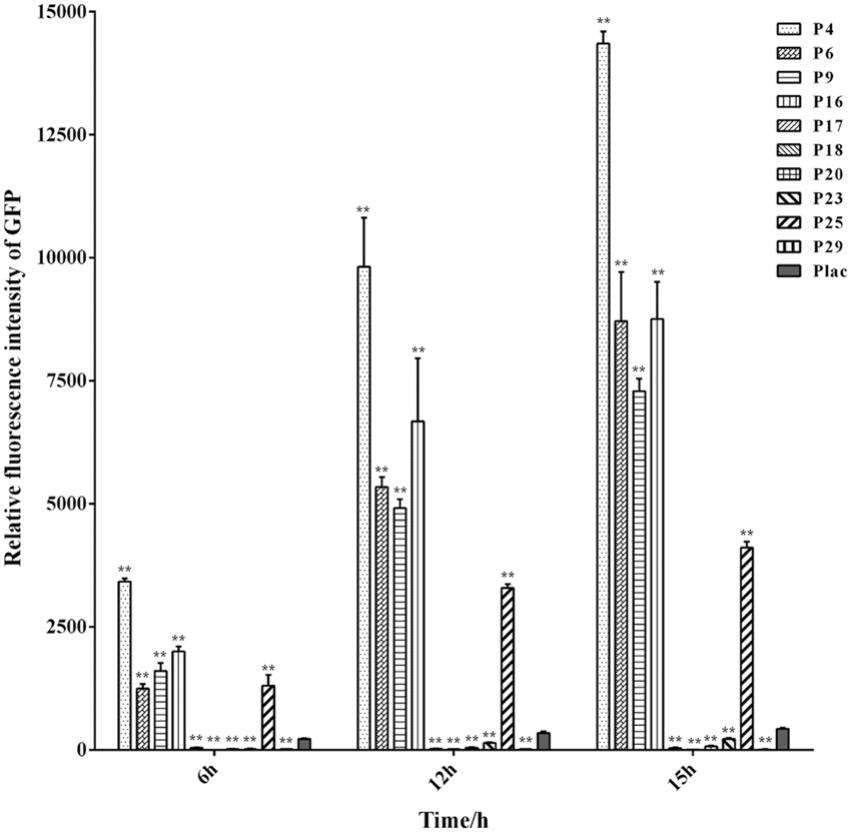
Characterization of the chosen promoters and *lac* promoter via GFP fluorescence intensity measurements. Expression of *gfp* gene under different promoters in *P. mendocina* NKU was quantified at different growth phases. The background expression was subtracted, and the relative fluorescence intensity was calculated by normalization against per OD_600_ of whole cells. Data represent the mean values ± standard deviations of triplicate measurements from three independent experiments. A Student’s *t*-test was performed between *lac* promoter and chosen promoters. * and ** indicate *P* < 0.05 and *P* < 0.01, respectively.

To further determine the expression levels of the selected endogenous promoters, relative fluorescence intensities were measured at three different growth phases. As shown in Fig. 3, the five strong promoters P4, P6, P9, P16 and P25 characterized by qPCR had also higher relative fluorescence intensities than *lac* promoter at any growth phase. P4 had the strongest relative fluorescence intensity among the 10 selected promoters, which showed a nearly 32-fold enhancement compared with *lac* promoter at the stationary phase. For each of the above five strong promoters, significant difference in the intensity of GFP fluorescence was observed at different growth stages, which was in agreement with the previous results on the unstable transcriptional levels of *gfp* measured by qPCR (Fig. S3). All of the results suggest that the expression levels of the five strong promoters might not be constant over the entire growth cycle (Fig. 3), The orders of promoter strength reflected by the real-time qPCR and GFP reporter were identical to the result obtained by RNA-seq (RPKM value), which demonstrated that the results of transcriptome sequencing analysis and functional validation experiments were highly consistent.

Interestingly, the relative transcriptional level of P25 was higher than that of P6, P9 and P16 (Fig. 2), but the relative fluorescence intensity of P25 was lower than that of P6, P9 and P16 (Fig. 3). The relative fluorescence intensities of the remaining promoters P17, P18, P20, P23 and P29 were lower than that of *lac* promoter at any growth phase, although P20 and P23 showed higher transcriptional levels than *lac* promoter. The observations suggest that the high transcriptional level of a gene might not necessarily lead to the high-level synthesis of this protein encoded by the gene. To maintain the consistency of translation initiation efficiency, in this study, the same RBS was introduced into upstream of reporter gene *gfp*. Among the reporter gene vectors, the distance between the predicted promoter sequences and RBS was different from each other, which may affect the efficiency of mRNA translation, possibly leading to the discrepancy between the transcriptional level and fluorescence intensity. For example, the distance between the predicted promoter sequences and RBS for P25 were longer than that for P6, P9 and P16. This may be the reason why P25 had higher transcriptional level, but lower fluorescence intensity than those of P6, P9 and P16. In addition, the differences in the spacer sequences between promoter and RBS may be the second reason for the different trends between the transcriptional level and fluorescence intensity.

**Figure 4:**
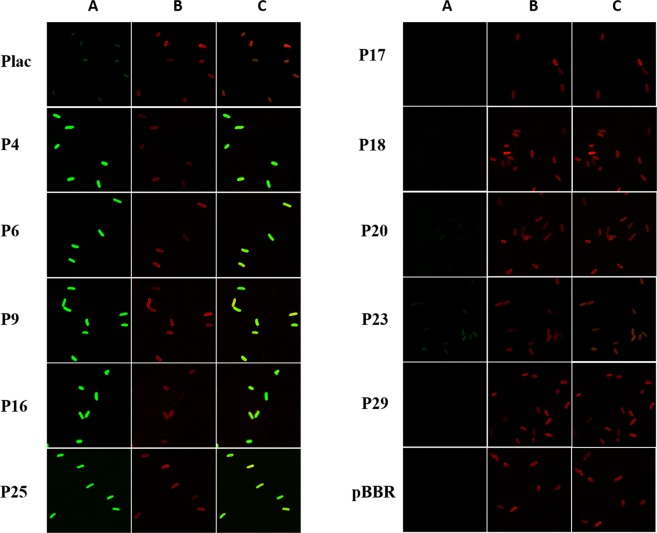
Characterization of the chosen promoters and *lac* promoter via confocal microscope. (**A**) Green fluorescence within the cell. (**B**) Outline of cell membrane by stain with FM4-64/L. (**C**) A and B merged together. All the images were taken at the same exposure condition.

When observed by confocal microscopy, cells expressing *gfp* under the control of P4, P6, P9, P16 and P25 produced more bright green fluorescence than the control cells with *gfp* expression under the control of *lac*. Cells produced weak green fluorescence when *gfp* expression was driven by P23 and P20. However, green fluorescence was not observed on the cells when expression of *gfp* was under the control of P17, P18 and P29 (Fig. 4). The results from confocal microscope matched well with that from GFP fluorescence intensity measurement.

In a previous study, a set of synthetic promoters, which is capable of stable and constitutive expression of downstream genes, was applied for calibrated heterologous gene expression in *P. putida* KT2440 using a mini-Tn7 delivery transposon vector that inserts the promoters into the genome of *P. putida*^28^. In another study, different inducible promoters were characterized by the construction of ProUSER-reporter vectors for use in *P. putida* KT2440, and the production of *p*-coumaric acid in *P. putida* KT2440 was enhanced by the use of selected inducible promoters for the optimization of pathway expression^29^. In this work, the five endogenous strong promoters P4, P6, P9, P16 and P25 were identified from *P. mendocina* NK-01 using a pipeline consisting of RNA-seq analysis and transcriptional level and fluorescence intensity measurements of a reporter gene *gfp*. So far, very little is known about the screening of strong promoters from the genus *Pseudomonas* using a RNA-seq-based strategy.

### Enhanced production of PHA by overexpressing *phaC* using the strong promoters in *P. mendocina* NKU

**Figure 5:**
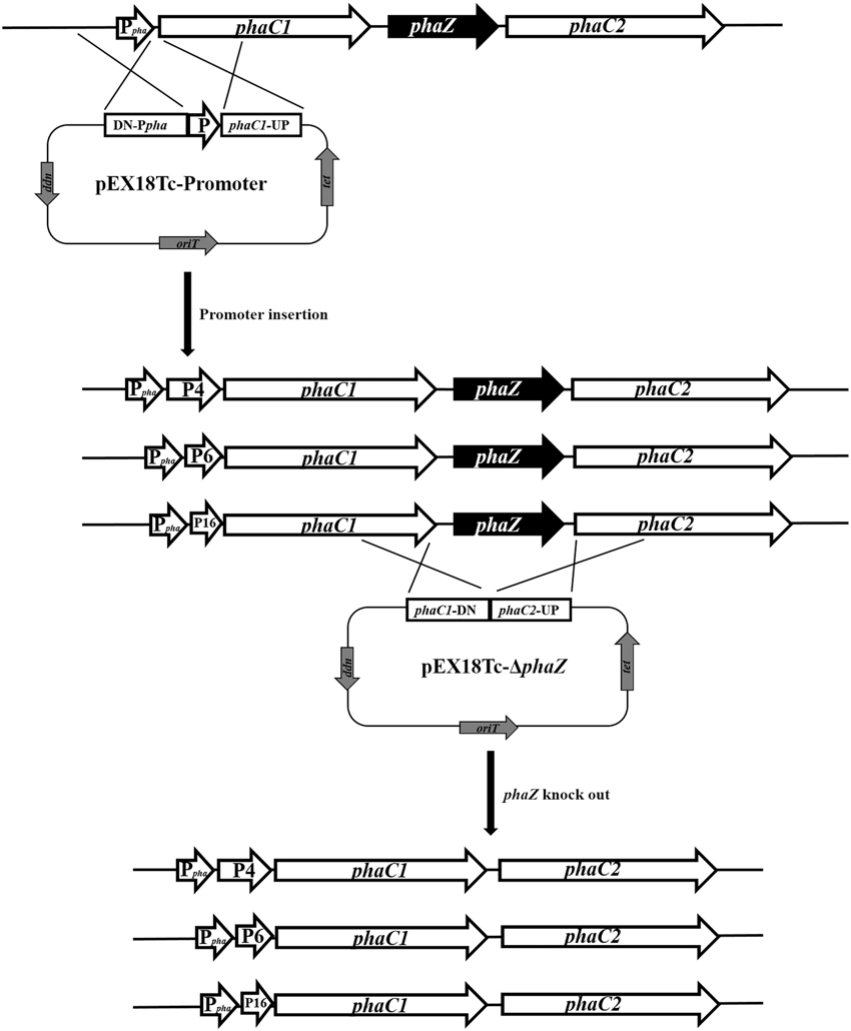
The construction schematic diagram for inserting the promoters into upstream of *phaC1* gene and for knockout of *phaZ* in the genome of *P. mendocina* NKU.

The mcl-PHA synthetic operon of *P. mendocina* NK-01 had been expounded in the earlier research. Our study has shown that PhaC1 is the main contributor to mcl-PHA synthesis in *P. mendocina* NK-01^23^. Consequently, overexpressing PHA synthase genes, especially the *phaC1* gene, may have a positive influence on mcl-PHA accumulation. The Standard European Vector Architecture Database (SEVA) has developed a series of plasmid vectors for metabolic engineering and synthetic biology in *Pseudomonas* and other gram-negative bacteria^30,31^. However, plasmid expression systems tend to be a burden on the bacteria, especially when multiple genes are needed to be co-expressed in a bacterium. In this work, the 3 endogenous strong promoters P4, P6 and P16 were selected for overexpressing PHA synthase genes by unmarked insertion of promoters upstream of the *phaC1* gene in the genome of *P. mendocina* NKU. This process did not leave any redundant sequences in the genome except the inserted promoter sequences (Fig. 5). This scarless genome editing strategy may confer some advantages over plasmid-borne overexpression of *phaC* genes.

**Figure 6:**
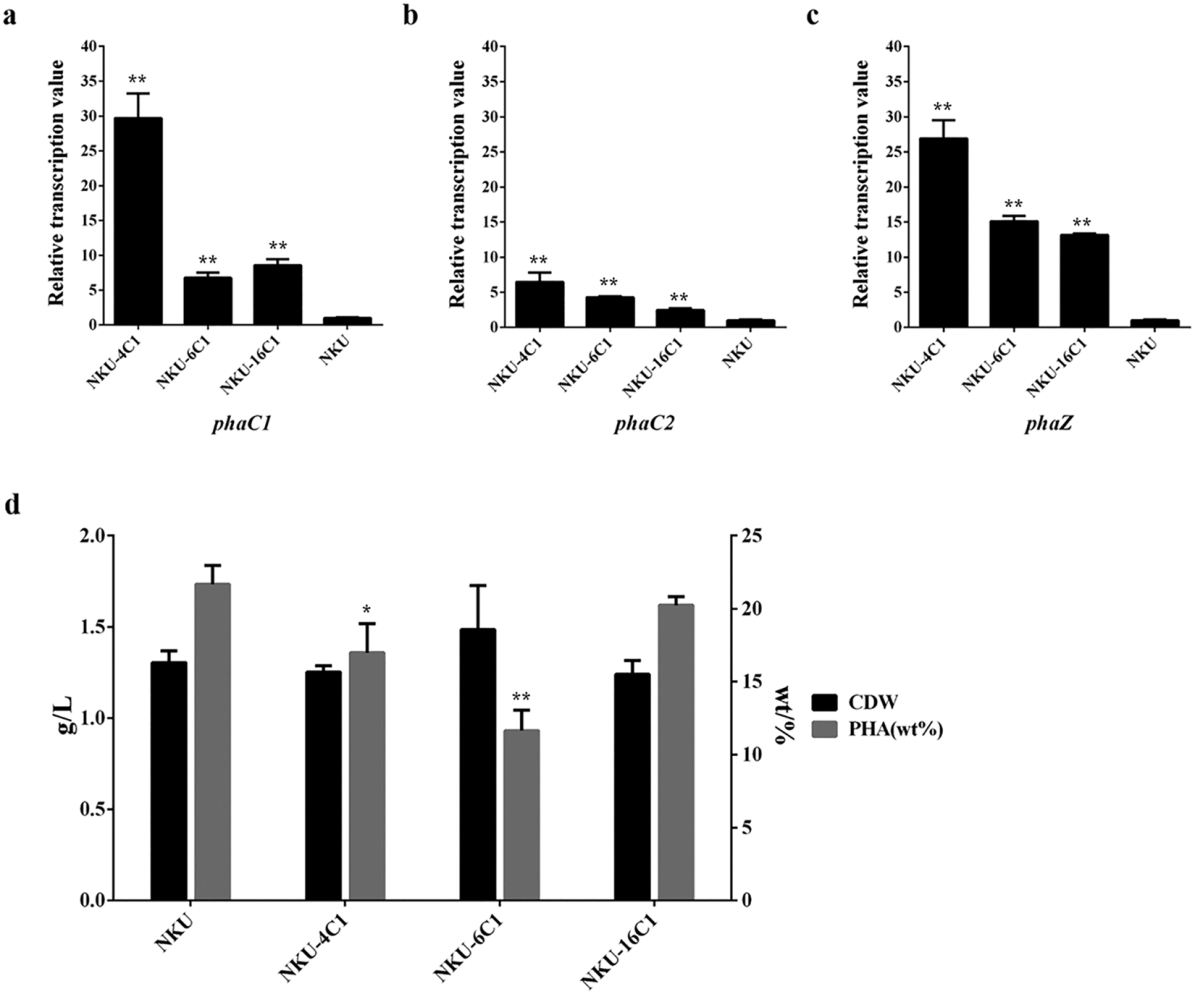
qPCR analysis and PHA fermentation results for the strains NKU-4C1, NKU-6C1, NKU-16C1 and NKU. Transcriptional levels of *phaC1* (**a**), *phaC2* (**b**) and *phaZ* (**c**) for the different strains. (**d**) Cell dry weight (CDW) and PHA production for the strains. Samples for qPCR were taken at 36 h of PHA fermentation. The transcriptional level for strain NKU was set as 1. wt% was defined as the ratio of PHA to CDW. Data represent the mean values ± standard deviations of triplicate measurements from three independent experiments. A Student’s *t*-test was performed between NKU and the mutants. * and ** indicate *P* < 0.05 and *P* < 0.01, respectively.

Through chromosomal insertion of the 3 endogenous strong promoters P4, P6 and P16, the transcriptional levels of *phaC1* and *phaC2* in the recombinant strains NKU-4C1, NKU-6C1 and NKU-16C1 were all improved compared with strain NKU. In particular, *phaC1* showed a more obvious improvement than *phaC2* (Fig. 6). This may be due to the fact that *phaC1* is closer to the inserted promoters than *phaC2*.

Moreover, the *phaZ* located between *phaC1* and *phaC2* had high transcriptional levels in the recombinant strains. Especially for NKU-6C1 and NKU-16C1, the transcriptional levels of *phaZ* were improved even more than *phaC1*, even though *phaZ* was far from the promoter in the gene cluster when compared to *phaC1*. This could because of that tight regulatory coupling between PHA polymerase activity and depolymerase activity may exist in this strain. It had been reported that single overexpression of PhaC may lead to an increase in the expression of PhaZ^32^. So *phaZ* showed a higher transcriptional level than *phaC1*, when PhaC and PhaZ were overexpressed simultaneously in a gene cluster. The PHA fermentation results showed that the PHA titers of NKU-4C1, NKU-6C1 and NKU-16C1 were all reduced, especially in NKU-6C1 (Fig. 6). These observations suggest that the overexpression of *phaZ* may lead to excessive synthesis of PHA depolymerase, and that the intracellularly accumulated PHA may be degraded by the depolymerase. The regulatory roles of PHA depolymerase in the synthesis of PHA were investigated previously by other researchers. For example, overexpression of PhaC2 alone in *P. putida* strain U was unable to accumulate higher amounts of PHA than in the wild-type strain, as a result of elevated PHA depolymerization in the late stage of PHA synthesis. A *phaZ*-inactive mutant of *P. putida* strain U, however, accumulated higher levels of PHA than the parental strain^32^. The mcl-PHA content of a *phaZ* knockout mutant of *P. putida* KT2442 (86 wt%, the ratio of PHA to CDW) was higher than that of wild-type strain (66 wt%) when using sodium octanoate as the carbon source^33^. However, the elimination of PHA depolymerase activity in *P. putida* KT2440 had little impact on the overall yield of PHA^34^. Both a *phaZ*-deficient mutant of *P. oleovorans* GPo1^35^ and two transposon-disrupted *phaZ* mutants of *P. resinovorans*^36^ did not show any substantial increase in PHA titer under various PHA synthesis conditions.

**Figure 7:**
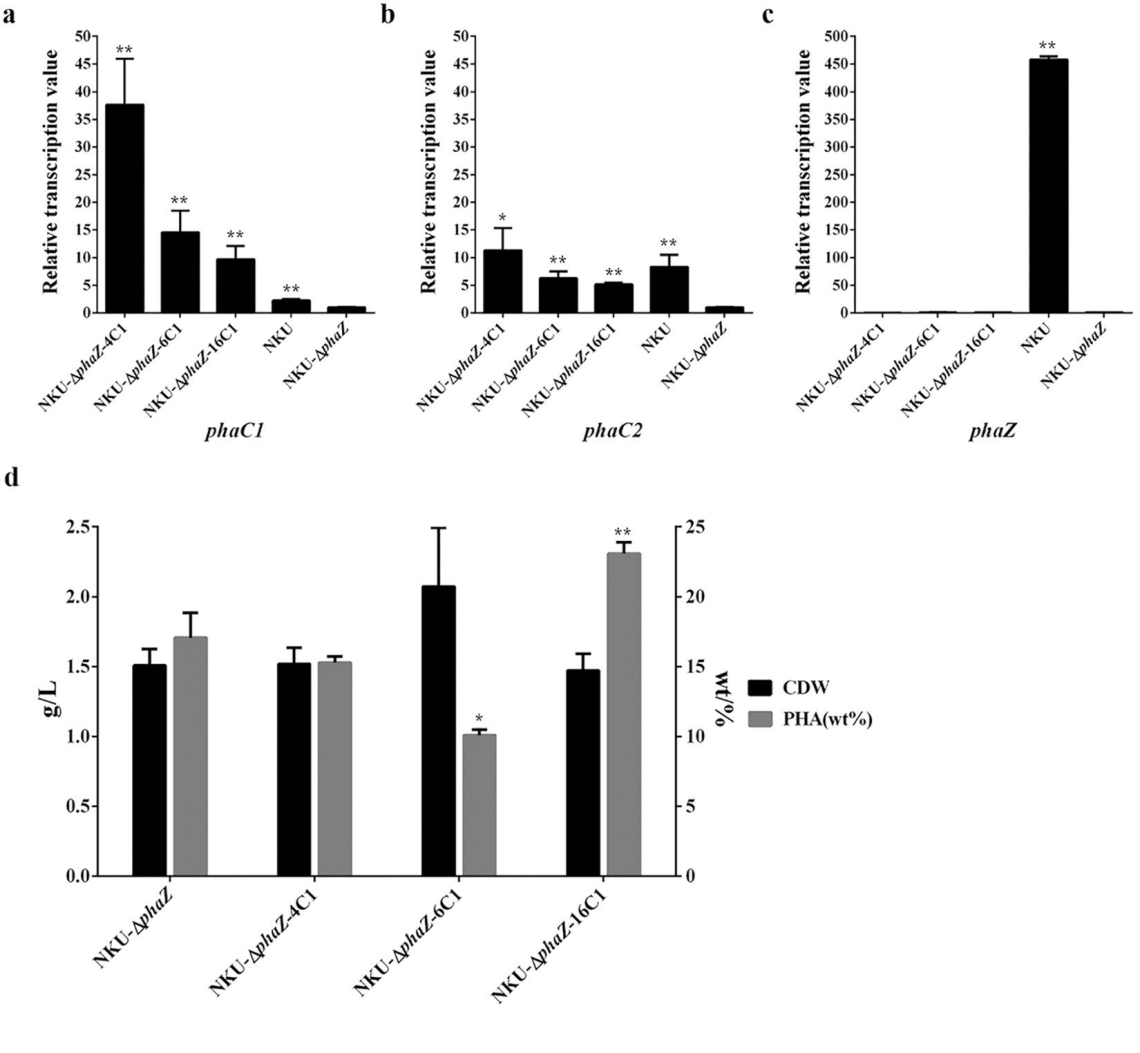
qPCR analysis and PHA fermentation results for the strains NKU-∆*phaZ*-4C1, NKU-∆*phaZ*-6C1, NKU-∆*phaZ*-16C1 and NKU-∆*phaZ*. Transcriptional levels of *phaC1* (**a**), *phaC2* (**b**) and *phaZ* (**c**) for the different strains. (**d**) CDW and PHA production for the strains. Samples for qPCR were taken at 36 h of PHA fermentation. The transcriptional level for strain NKU-∆*phaZ* was set as 1. Data represent the mean values ± standard deviations of triplicate measurements from three independent experiments. A Student’s *t*-test was performed between NKU-∆*phaZ* and other mutants. * and ** indicate *P* < 0.05 and *P* < 0.01, respectively.

In this work, we attempt to improve the yield of PHA by the construction of *phaZ* knockout mutants. However, the PHA titer of strain NKU-*phaZ* was decreased by 4 wt% compared with strain NKU from 21 to 17 wt% (Fig. 7), indicating that knockout of *phaZ* cannot improve the yield and molecular weight of mcl-PHA in *P. mendocina* NK-01. Surprisingly, PHA synthesized by all *phaZ* knockout mutants had lower molecular weights than PHA synthesized by the parent strain NKU, with an exception of NKU-*phaZ*-6C1 (Table S2). mcl-PHAs synthesized by *P. mendocina* NKU and its mutant strains were mainly composed of three different monomers, i.e., 3-hydroxyoctanoate, 3-hydroxydecanoate and 3-hydroxydodecanoate, as shown by GC-MS analysis (Figs S4, S5). The monomer composition ratios of the mcl-PHAs had not obvious changes for the mutants compared with NKU (Table S3).

The relative transcriptional values of *phaC1* and *phaC2* in strain NKU-*phaZ*-4C1, NKU-*phaZ*-6C1 and NKU-*phaZ*-16C1 were all improved compared with NKU-*phaZ*, (Fig. 7). Interestingly, the relative transcriptional values of *phaC1* and *phaC2* in strain NKU-*phaZ*-16C1 were the lowest among the above three strains, while the PHA titer of strain NKU-*phaZ*-16C1 was the highest among the above three strains. The PHA titer of strain NKU-*phaZ*-4C1 was similar to that of strain NKU-*phaZ*. Compared with strain NKU-*phaZ*, the PHA titer of strain NKU-*phaZ*-6C1 was reduced by 7%, and the PHA titer for strain NKU-*phaZ*-16C1 were improved by 6% to 23 wt% (Fig. 7). These results indicated that the expression level of *phaC* was not positively related to the PHA titer in strain NK-01. In future studies, the optimal expression of *phaC* may be required for obtaining the highest PHA yield in strain NK-01. It should be noted that the native RBS sequence of the *phaC* gene was unchanged when the strong endogenous promoters were inserted into upstream of the *phaC* operon in the genome of *P. mendocina*. In *P. mendocina*, the native RBS sequence may be optimal for the translational initiation of the *phaC* operon. RBS can serve as an important regulatory element for translational initiation and thus obviously affect the gene expression level^37,38^. Only using strong promoters may not obtain the optimal expression levels. For this study, the optimization of the RBS sequence coupled with the screening of endogenous strong promoters may be required for the optimal PHA synthase gene expression.

The relative transcriptional values of *phaC1* and *phaC2* for the different mutant strains at 12 h and 24 h of mcl-PHA fermentation were also measured, respectivly. As expected, the strains NKU-*phaZ*-4C1, NKU-*phaZ*-6C1 and NKU-*phaZ*-16C1 showed higher transcriptional levels for *phaC1* and *phaC2* than NKU-*phaZ* at 12 h, however, no significant differences were observed among the above three strains (Fig. S6). At 24 h, the relative transcriptional values of *phaC1* for the above three strains were also improved compared with NKU-*phaZ*, and NKU-*phaZ*-4C1 had the highest transcriptional level among the three strains (Fig. S6). Surprisingly, the transcriptional levels for *phaC2* at 24 h had not obvious increase for NKU-*phaZ*-4C1, NKU-*phaZ*-6C1 and NKU-*phaZ*-16C1 compared with NKU-*phaZ* (Fig. S6). The strain NKU-*phaZ*-16C1 showed the lowest transcriptional levels for *phaC1* and *phaC2* in any timepoints (Fig. 7 and Fig. S6). These results indicated that the transcriptional levels of *phaC1* and *phaC2* for the mutant strains were not constant during the PHA fermentation. The changes in the transcriptional levels of the PHA synthase genes over the PHA fermentation period may contribute to lower increase in the PHA yield.

Since a complex metabolic pathway is involved in the synthesis of PHA from glucose, there are many important factors to influence the efficiency of PHA synthesis, including the Entner-Doudoroff pathway, the flux of acetyl-CoA, the fatty acid *de novo* synthesis pathway, and the availability of the PHA synthesis^39–41^. Modification of a few factors may not have an obvious influence for the improvement of PHA synthesis.

In this work, all *phaZ* knockout mutants showed a decrease in the relative transcriptional levels of *phaC1* and *phaC2* compared with their corresponding strains without deletion of *phaZ*. Compared with strain NKU-*phaZ*, the reduction in the PHA titer was observed with strain NKU-*phaZ*-4C1 and NKU-*phaZ*-6C1, while the PHA titer was improved for strain NKU-*phaZ*-16C1 (Fig. 7). These results suggest that PhaZ is not only involved in PHA degradation but also acts as an important role in PHA synthesis in *P. mendocina* NK-01. A previous study has shown that PhaZ may play a crucial role in the turnover of mcl-PHA under starvation conditions in *P. putida* KT2442^42^. Rational tuning of the transcriptional activity of PHA synthase and depolymerase would be a feasible approach for the optimization of PHA production in strain NK-01. Therefore, we believe that the screened endogenous strong promoters have the potential to be applied for overexpression of PHA synthesis pathway genes to improve the production of PHA in *P. mendocina* NK-01.

Promoter engineering such as the screening of strong promoters has been widely applied for metabolic pathway engineering to improve the yield of many industrial products. However, in many cases, the exogenous promoters may not be compatible with the native gene expression systems in *P. mendocina* NK-01. A previous study in our lab showed that the PHA yield had an obvious decrease after the overexpression of PHA synthase genes using an exogenous strong promoter J23119 in *P. putida* KT2440 (unpublished data). This also shown it’s not that the more of PhaC expression was, the higher of mcl-PHA yield could get. In this study, we didn’t select a very strong exogenous promoter as the reference and the commonly used *lac* promoter^43,44^ has been used as the control in the transcriptional activity assays to screen appropriate endogenous strong promoters. Compared with the *lac promoter*, the screened endogenous promoters P4, P6 and P16 showed higher transcriptional activity and fluorescence intensity. Therefore, we tested the ability of the screened endogenous promoters to improve the production of mcl-PHA by overexpressing PHA synthase in *P. mendocina* NK-01. Future work is needed to screen more suitable promoters and optimize the PHA biosynthetic pathway to further improve the mcl-PHA production, not only via overexpression of PHA synthase genes. And the screened endogenous promoters can also be applied to enhance the biosynthesis of AO which is another product synthesized by NK-01 from glucose. The use of endogenous promoters may be a feasible method for the optimization of the expression of the synthetic pathway genes, and this strategy could be potentially utilized for enhanced production of other valuable bio-based products.

## Conclusions

In this study, we used the screened endogenous promoters to improve the production of mcl-PHA by overexpressing PHA synthase in *P. mendocina* NK-01. The use of endogenous promoters may be a feasible method for the optimization of the expression of the synthetic pathway genes, and this strategy could be potentially utilized for enhanced production of other valuable bio-based products.

## Materials and Methods

### Bacterial strains, plasmids, and growth conditions

*E. coli* DH5α was used for plasmid construction. *E. coli* S17-1 was used for conjugal transfer. *P. mendocina* NK-01 is a chloramphenicol resistant, mcl-PHA-producing strain and is deposited in the China Center for Type Culture Collection (CCTCC, accession no. CCTCC M 208005). *P. mendocina* NKU, an *upp*-deficient strain of *P. mendocina* NK-01^25^, was used as the target strain. An *E. coli*-*Pseudomonas* shuttle vector pBBR1MCS-2 was used to verify the strengths of 10 endogenous promoters from *P. mendocina* NK-01 using a reporter gene *gfp*. A suicide plasmid pEX18Tc-*upp* was used for promoter integration or *phaZ* knockout. All strains and plasmids used in this study are listed in Table 2.

**Table 2:** Strains and plasmids used in this study.

Strains and plasmids	Description	Source or reference
**Strains**		
*Pseudomonas mendocina* NKU	PHA_MCL_ producing strain; Cm^R^; preserved in our laboratory; carrying an in-frame deletion in the *upp* gene; starting strain for engineering	This laboratory
*P. mendocina* NKU pBBR	NKU derivative containing pBBR1MCS-2; Cm^R^; Km^R^	This work
*P. mendocina* NKU-P*lac*	NKU derivative containing pBBR1MCS-*gfp*; Cm^R^; Km^R^	This work
*P. mendocina* NKU-P4	NKU derivative containing pBBR1MCS-P4-*gfp*; Cm^R^; Km^R^	This work
*P. mendocina* NKU-P6	NKU derivative containing pBBR1MCS-P6-*gfp*; Cm^R^; Km^R^	This work
*P. mendocina* NKU-P9	NKU derivative containing pBBR1MCS-P9-*gfp*; Cm^R^; Km^R^	This work
*P. mendocina* NKU-P16	NKU derivative containing pBBR1MCS-P16-*gfp*; Cm^R^; Km^R^	This work
*P. mendocina* NKU-P17	NKU derivative containing pBBR1MCS-P17-*gfp*; Cm^R^; Km^R^	This work
*P. mendocina* NKU-P18	NKU derivative containing pBBR1MCS-P18-*gfp*; Cm^R^; Km^R^	This work
*P. mendocina* NKU-P20	NKU derivative containing pBBR1MCS-P20-*gfp*; Cm^R^; Km^R^	This work
*P. mendocina* NKU-P23	NKU derivative containing pBBR1MCS-P23-*gfp*; Cm^R^; Km^R^	This work
*P. mendocina* NKU-P25	NKU derivative containing pBBR1MCS-P25-*gfp*; Cm^R^; Km^R^	This work
*P. mendocina* NKU-P29	NKU derivative containing pBBR1MCS-P29-*gfp*; Cm^R^; Km^R^	This work
*P. mendocina* NKU-4C1	NKU derivative carrying an P4 promoter insertion into the upstream of *phaC1* gene	This work
*P. mendocina* NKU-6C1	NKU derivative carrying an P6 promoter insertion into the upstream of *phaC1* gene	This work
*P. mendocina* NKU-16C1	NKU derivative carrying an P16 promoter insertion into the upstream of *phaC1* gene	This work
*P. mendocina* NKU-∆*phaZ*-4C1	NKU-4C1 derivative carrying an in-frame deletion in the *phaZ* gene	This work
*P. mendocina* NKU-∆*phaZ*-6C1	NKU-6C1 derivative carrying an in-frame deletion in the *phaZ* gene	This work
*P. mendocina* NKU-∆*phaZ*-16C1	NKU-16C1 derivative carrying an in-frame deletion in the *phaZ* gene	This work
*P. mendocina* NKU-∆*phaZ*	NKU derivative carrying an in-frame deletion in the *phaZ* gene	This work
*Escherichia coli* DH5α	F^−^, φ80d*lac*ZΔM1, Δ(*lacZYA-argF*)U169, *deoR*, *recA*1, *endA*1, *hsdR*17(r_k_^−^, m_k_^+^), *phoA*, *supE*44, λ^−^*thi*-1, *gyrA*96, *relA*1	This laboratory
*E. coli* S17-1	*recA*; harbors the *tra* genes of plasmid RP4 in the chromosome; *proA thi-1*	This laboratory
**Plasmids**		
pBBR1MCS-2	Broad host range; expression plasmid; Kan^r^; P_*lac*_, *mob*	^27^
pBBR1MCS-*gfp*	pBBR1MCS-2 derivative containing *gfp* gene	This work
pBBR1MCS-P4-*gfp*	pBBR1MCS-2 derivative containing P4 promoter and *gfp* gene	This work
pBBR1MCS-P6-*gfp*	pBBR1MCS-2 derivative containing P6 promoter and *gfp* gene	This work
pBBR1MCS-P9-*gfp*	pBBR1MCS-2 derivative containing P9 promoter and *gfp* gene	This work
pBBR1MCS-P16-*gfp*	pBBR1MCS-2 derivative containing P16 promoter and *gfp* gene	This work
pBBR1MCS-P17-*gfp*	pBBR1MCS-2 derivative containing P17 promoter and *gfp* gene	This work
pBBR1MCS-P18-*gfp*	pBBR1MCS-2 derivative containing P18 promoter and *gfp* gene	This work
pBBR1MCS-P20-*gfp*	pBBR1MCS-2 derivative containing P20 promoter and *gfp* gene	This work
pBBR1MCS-P23-*gfp*	pBBR1MCS-2 derivative containing P23 promoter and *gfp* gene	This work
pBBR1MCS-P25-*gfp*	pBBR1MCS-2 derivative containing P25 promoter and *gfp* gene	This work
pBBR1MCS-P29-*gfp*	pBBR1MCS-2 derivative containing P29 promoter and *gfp* gene	This work
pEX18Tc-*upp*	pEX18Tc derivative, carrying a copy of *upp* gene of *P. mendocina* NKU	This laboratory
pEX18Tc-Δ*phaZ*	pEX18Tc-*upp* derivative, carrying the up- and downstream regions of *phaZ* gene, used for deletion of the *phaZ* gene	This work
pEX18Tc-P4	pEX18Tc-*upp* derivative, used for insertion of P4 promoter in front of *phaC1*	This work
pEX18Tc-P6	pEX18Tc-*upp* derivative, used for insertion of P6 promoter in front of *phaC1*	This work
pEX18Tc-P16	pEX18Tc-*upp* derivative, used for insertion of P16 promoter in front of *phaC1*	This work

Luria-Bertani (LB) agar plates (10 g/L tryptone, 5 g/L yeast extract, 5 g/L NaCl and 2 g/L agar) was used to activate bacteria that stored at −80 °C. *E. coli* strains were cultivated in LB medium (10 g/L tryptone, 5 g/L yeast extract and 5 g/L NaCl)^45^ at 37 °C and 180 rpm on a rotary shaker. *P. mendocina* strains were cultivated in LB medium, nutrient-rich (NR) medium (10 g/L yeast extract, 10 g/L peptone, 5 g/L beef extract power and 5 g/L (NH_4_)_2_SO_4_) or PHA fermentation medium (20 g/L glucose, 9.58 g/L Na_2_HPO_4_·12H_2_O, 2.65 g/L KH_2_PO_4_, 0.20 g/L MgSO_4_ and 1 mL trace element solution) at 30 °C^22^. For characterization of the endogenous promoters, *P. mendocina* strains were cultured in 100 mL LB medium in a 500 mL un-baffled flask and cultivated at 30 °C and 180 rpm on a rotary shaker. When necessary, media were supplemented with kanamycin (Kan, 50 μg/mL), tetracycline (Tc, 25 μg/mL), chloramphenicol (Cm, 170 μg/mL) or 5-fluorouracil (5-FU, 20 μg/mL).

### RNA-seq and promoter prediction

The cultures of *P. mendocina* NK-01 in PHA fermentation medium were sampled every 6 h. Then, the samples were mixed equally for total RNA extraction using an RNApure bacteria kit (Cwbio, Beijing, China). Qualified samples with RIN larger than 8 were submitted to BGI (Shenzhen, China) for RNA-seq analysis.

The expression levels of predicted genes were quantified in terms of RPKM as previously defined^26,46^. Firstly, the RPKM values were used to rank the gene transcription levels. The upstream regions of highly active genes with high RPKM values were preliminarily selected for promoter prediction. Next, a web-based platform for promoter prediction (Neural Network Promoter Prediction, http://www.fruitfly.org/seq_tools/promoter.html) was used to identify whether the selected upstream regions contain consensus sequence of a promoter. Finally, 10 candidate sequences that can be precisely forecasted to a promoter sequence were chosen for further studies.

### Construction of reporter gene vectors

Reporter gene vectors were constructed for the measurement of the strengths of 10 promoters. Each promoter sequence and *gfp* gene were amplified by PCR, respectively, from genomic DNA of *P. mendocina* NK-01 and pWH1520-*gfp*. Then, a fusion fragment including a promoter sequence and *gfp* gene was obtained by overlapping PCR and inserted into pBBR1MCS-2 in an opposite direction relative to *lac* promoter on the plasmid using homologous recombination. The same RBS sequence (AGGAGG) was incorporated into upstream of *gfp* gene by PCR during the construction of all reporter gene vectors. A control vector expressing *gfp* gene with the above RBS sequence under the control of *lac* promoter was also constructed with pBBR1MCS-2.

### Real-time quantitative PCR for *P. mendocina* containing various reporter gene vectors

Cells from 1 mL of LB culture were collected at 6, 12, and 15 h. Total RNA was extracted using a commercial RNA pure Bacteria Kit (Cwbio, Beijing, China). After the removal of DNA contamination, cDNA was synthesized by reverse transcription using a HiScript II Q RT SuperMix (Vazyme, Nanjing, China). Real-time PCR was performed with FastStart Universal SYBR Green Master (Roche, Basel, Switzerland) on a StepOnePlus^TM^ real-time PCR system (Applied Biosystems, Foster City, CA, USA). PCR conditions were as follows: pre-incubation at 95 °C for 10 min, followed by 40 cycles of denaturation at 95 °C for 30 s, annealing at 55 °C for 30 s and extension at 72 °C for 20 s. Triplicates were used for all analysis. Relative gene expression levels were calculated against the 16S rDNA gene as the internal reference using the 2^−ΔΔCt^ method^11,47^. The relative promoter activity (RPA) was calculated by normalization against that of *lac* promoter.

### Fluorescence measurement and confocal microscopy for *P. mendocina* containing various reporter gene vectors

The quantitative measurement of GFP was performed via a microplate reader. Cells from 200 μL of LB culture were collected at 6, 12 and 15 h, washed thrice with phosphate-buffered saline (PBS) buffer (pH 7.4), and resuspended (OD_600_ = 0.5) in 200 μL of PBS buffer. The relative fluorescence intensity was measured with an Enspire Reader System (Perkinelmer, Waltham, USA) at an excitation wavelength of 395 nm and an emission wavelength of 509 nm. The cell optical density at 600 nm was determined using a UV-1800 spectrophotometer (Shimadzu, Kyoto, Japan). Relative fluorescence intensity was calculated by normalization against per OD_600_ of whole cells. The fluorescence signal of *P. mendocina* NKU harboring pBBR1MCS-2 was set as background and was subtracted from the overall fluorescence.

Qualitative observation for the expression of GFP was carried out through the confocal microscopy in a visual form under the same imaging parameters. Cells were harvested after incubation for 12 h in LB medium, washed thrice with PBS buffer (pH 7.4), and resuspended in 400 μL of PBS buffer. Next, cells were stained with 10 μM FM4-64/L for 15 min in the dark and then fixed with 2% glycerol on a slide. The fluorescence image was acquired using a confocal laser scanning microscope LSM710 (Zeiss, Oberkochen, Germany) fitted with a Zeiss 100 × 10 numerical aperture objective lens using the argon laser at 395 nm for GFP excitation. The master gain was 800 and the laser intensity was 2.0% for GFP imaging.

### Construction of *P. mendocina* mutant strains

The *P. mendocina* mutants with promoter insertion and *phaZ* deleted were constructed based on a scarless genome editing strategy^25^ using the suicide plasmid in combination with *upp* as a counter-selectable marker. The *upp* knockout mutant strain *P. mendocina* NKU was constructed as described previously^25^. Different promoter sequences were fused independently with the upstream and downstream homologous arms of *phaC1* by overlapping PCR, and the fusion fragments were incorporated independently into the suicide plasmid pEX18Tc-*upp* to generate the gene targeting vectors. The constructed vectors were transformed independently into *P. mendocina* NKU via conjugal transfer with *E. coli* S17-1 as the vector donor strain^25^.

Since the introduced plasmids cannot be replicated autonomously in *P. mendocina*, they have to integrate via homologous recombination into the chromosome. The single-crossover recombinants were screened by incubating at 30 °C for 24 h on LB agar plates supplemented with 25 μg/mL Tc and 170 μg/mL Cm. Then the selected recombinants were incubated at 30 °C for 24 h in LB medium. To further screen the double-crossover recombinants, the culture broths that had been diluted to 10^−2^ were spread on LB agar plates supplemented with 20 μg/mL 5-FU. The selected recombinants showing 5-FU^r^ and Tc^s^ were further checked by PCR. All the constructed mutants were validated by DNA sequencing. Furthermore, the *phaZ* gene of the constructed mutants was deleted from the genome using the above genome editing strategy. All primers used for vector construction and mutant validation are listed in Table S4. The transcriptional level of the *phaC* operon in the mutant strains was detected by real-time PCR as described above.

### Shake-flask fermentation for mcl-PHA production by *P. mendocina*

PHA production by *P. mendocina* was achieved with a two-step fermentation process, including the stages of cell proliferation and PHA synthesis. Overnight culture (1%, v/v) was inoculated into 100 mL of NR medium^22^ in a 500 mL un-baffled flask and then incubated at 30 °C and 180 rpm on a rotary shaker for 24 h. Bacterial cells were harvested by centrifugation at 2,500 × g and 4 °C and resuspended in 1 mL PBS buffer. Next, the seed culture was inoculated into 100 mL of fermentation medium, pH 7.0^22^ in a 500 mL un-baffled flask and cultivated at 30 °C and 180 rpm on a rotary shaker for 36 h. After fermentation, the culture broth was centrifuged at 4 °C and 13,000 × g for 20 min to collect bacterial cells. Cells were lyophilized for 24 h and weighed. Finally, PHA was extracted from lysed cells with chloroform at a rate of 100 mL chloroform g^−1^ cells at room temperature for 2 days^21,22^. The extract containing PHA was filtered to remove cellular debris by the Whatman filter paper and then concentrated by a vacuum rotary evaporator. A 40-fold volume of pre-cooled methanol was added to precipitate PHA overnight. PHA was weighed after being dried at room temperature to remove all residual solvent^21,22^.

### Gel permeation chromatography (GPC) and gas chromatograph/mass spectrometry (GC/MS)

The molecular weight of PHA was estimated by GPC (Alltech, USA) with a K-804 gel column (Shodex, Japan) and a SFD differential refractive index detector (Schambeck, Germany) according to the previously established procedure^23^.

The monomer composition of PHA was determined by GC/MS analysis using an Agilent Technologies 7890A-5975C (Agilent Technologies, Palo Alto, CA, USA). Firstly, 5 mg of PHA sample was dissolved in 2 mL CHCl_3_ and subjected to methanolysis in the presence of 0.3 mL H_2_SO_4_ and 1.7 mL CH_3_OH at 100 °C for 140 min in order to obtain the corresponding 3-hydroxyalkanoic methyl esters. The produced monomers were further identified by comparing their GC and MS spectra with that of the authentic standards in GC/MS analysis. The detailed procedures for GC/MS analysis are described in Guo *et al*.^21^.

## Supplementary information

Supplementary Info

## Data Availability

The RNA-seq data used in this study are available in the Sequence Read Archive (SRA) repository with the BioProject accession number PRJNA514902, BioSample accession number SAMN10736309 and SRA accession number SRR8437825.
