# Supplementary material for: Screening of endogenous strong promoters for enhanced production of medium-chain-length polyhydroxyalkanoates in Pseudomonas mendocina NK-01
Source: Sci Rep. 2019 Feb 12;9:1798. doi: 10.1038/s41598-019-39321-z (PMC6372614; doi:10.1038/s41598-019-39321-z)
Supplement: Supplementary file 1 — Supplementary Info [file 41598_2019_39321_MOESM1_ESM.pdf]

Supplemental materials

Screening of endogenous strong promoters for enhanced production of medium-chain-length polyhydroxyalkanoates in *Pseudomonas mendocina* NK-01

Fengjie Zhao,[1] Xiangsheng Liu,[1] Annie Kong,[1] Yuxin Zhao,[1] Xu Fan,[1] Ting Ma,[1] Weixia Gao,[2]* Shufang Wang,[2]* Chao Yang[1]*

[1]Key Laboratory of Molecular Microbiology and Technology for Ministry of Education, Nankai University, Tianjin 300071, China

[2]State Key Laboratory of Medicinal Chemical Biology, Nankai University, Tianjin 300071, China

Correspondence to:

Weixia Gao

Tel./Fax: +86 22 23503753

E-mail: watersave@126.com

Shufang Wang

Tel./Fax: +86 22 23503753

E-mail: wangshufang@nankai.edu.cn

Chao Yang

Tel./Fax: +86 22 23503866

E-mail: yangc20119@nankai.edu.cn

Table S1 The top 30 genes ranked by transcriptional level based on their RPKM values in RNA-seq analysis

Table S2 The molecular weights of mcl-PHAs synthesized by *P. mendocina* NKU and its mutants

Table S3 The monomer composition ratios of mcl-PHA synthesized by *P. mendocina* NKU and its mutants

Table S4 Primers used in this study

Fig. S1 Cloned promoter sequences and predicted promoter sequences

Fig. S2 The growth curves of all the constructed strains for promoter characterization

Fig. S3 Transcriptional levels of the five strong promoters and *lac* promoter in different growth phases

Fig. S4 GC-MS analysis for the monomer compositions of mcl-PHA synthesized by *P. mendocina* NKU and its mutants

Fig. S5 The molecular structure of mcl-PHA produced by *P. mendocina* NK-01

Fig. S6 Transcriptional levels of *phaC1* and *phaC2* for the different mutant strains at 12 h and 24 h.

Table S1 The top 30 genes ranked by transcriptional level based on their RPKM values in RNA-seq analysis

Gene ID	Productions	Promoters	RPKM
MDS_4427	hypothetical protein	P1	36681.95
MDS_r003	16S ribosomal RNA	P2	33511.23
MDS_1604	hypothetical protein	P3	29531.20
MDS_2118	hypothetical protein	P4	24593.43
MDS_1524	anti sigma-E protein,RseA	P5	24300.80
MDS_0756	hypothetical protein	P6	24066.04
MDS_2424	general stress protein	P7	19061.06
MDS_3781	hypothetical protein	P8	17212.90
MDS_3450	hypothetical protein	P9	16182.31
MDS_3307	hypothetical protein	P10	15688.80
MDS_4616	hypothetical protein	P11	14094.26
MDS_1176	UspA domain-containing protein	P12	13610.24
MDS_1400	hypothetical protein	P13	13273.27
MDS_2117	hypothetical protein	P14	10945.60
MDS_3417	glyoxalase/bleomycin resistance protein/dioxygenase	P15	9162.63
MDS_2531	alcohol dehydrogenase	P16	8949.33
MDS_0957	ribosomal protein S30P / sigma 54 modulation protein	P17	8412.46
MDS_0563	poly(hydroxyalkanoate) granule-associated protein	P18	8222.26
MDS_2561	DNA-binding transcriptional activator	P19	7903.00
MDS_1497	17 kDa surface antigen/outer membrane lipoprotein	P20	7267.13
MDS_3574	PfpI family intracellular peptidase	P21	7239.34
MDS_4560	hypothetical protein	P22	7213.88
MDS_1161	arginine/ornithine antiporter	P23	6935.83
MDS_2084	hypothetical protein	P24	6818.40
MDS_4257	outer membrane protein W	P25	6637.68
MDS_1159	ornithine carbamoyltransferase	P26	6455.59
MDS_2628	OmpF family protein	P27	5967.23
MDS_0564	poly(hydroxyalkanoate) granule-associated protein	P28	5905.14
MDS_3908	transport-associated protein	P29	5888.12
MDS_2110	transglutaminase domain-containing protein	P30	5796.17

Table S2 The molecular weights of mcl-PHAs synthesized by *P. mendocina* NKU and its mutants

Strains	M_w/ ×10^4 Da	M_n/ ×10^4 Da	M_w/M_n
NKU	25.2	14.3	1.76
NKU-Δ*phaZ*	22.5	15.8	1.42
NKU-Δ*phaZ*-4C1	18.8	13.3	1.41
NKU-Δ*phaZ*-6C1	29.1	15.1	1.93
NKU-Δ*phaZ*-16C1	17.5	11.7	1.50

Table S3 The monomer composition ratios of mcl-PHA synthesized by *P. mendocina* NKU and its mutants

Strains	Monomer composition (%)		
	**C8**	**C10**	**C12**
NKU	17.87	77.84	4.29
NKU-Δ*phaZ*	16.27	79.35	4.38
NKU-Δ*phaZ*-4C1	19.38	74.39	6.23
NKU-Δ*phaZ*-6C1	22.13	73.41	4.46
NKU-Δ*phaZ*-16C1	15.11	79.86	5.03

Table S4 Primers used in this study

Primers	Description	Source or literature
P4-F	TATAGGGCGAATTG**GAGCTC**GGTACCGAGGTTTCCTGTGGTGAGCA	This work
P4-R	ATGAATTCGCCTCCTGAATTCGTGGGCGTGTCAGCTTGG	This work
gfp-P4-F	CACGCCCACGAATTCAGGAGGCGAATTCATGAGTAAAGGAGAAGAAC	This work
P6-F	TATAGGGCGAATTG**GAGCTC**GGTACCGGCTGAAGCGGCCTCCT	This work
P6-R	ATGAATTCGCCTCCTGAATTCGTTGGGGAGTAATAAGTGGCTTC	This work
gfp-P6-F	CTCCCCAACGAATTCAGGAGGCGAATTCATGAGTAAAGGAGAAGAAC	This work
P9-F	TATAGGGCGAATTG**GAGCTC**GGTACCCCTGCTGCGCCTTTCACAAGAC	This work
P9-R	ATGAATTCGCCTCCTGAATTCAGCGGGGTTGTGGTTGGGTATG	This work
gfp-P9-F	AACCCCGCTGAATTCAGGAGGCGAATTCATGAGTAAAGGAGAAGAAC	This work
P16-F	TATAGGGCGAATTG**GAGCTC**GGTACCATCCGAGGGATACGGGCCTTGTC	This work
P16-R	ATGAATTCGCCTCCTGAATTCGAGCTTGATCGCGGATTAACAC	This work
gfp-16-F	ATGAATTCGCCTCCTGAATTCGAGCTTGATCGCGGATTAACAC	This work
P17-F	TATAGGGCGAATTG**GAGCTC**GGTACCGCCAGATCGACTGAAGGCACAAG	This work
P17-R	ATGAATTCGCCTCCTGAATTCGTTGCCTTGTGTAAGAGACG	This work
gfp-17-F	CAAGGCAACGAATTCAGGAGGCGAATTCATGAGTAAAGGAGAAGAAC	This work
P18-F	TATAGGGCGAATTG**GAGCTC**GGTACC AGCTTCAAGCGGCAAGCCAC	This work
P18-R	ATGAATTCGCCTCCTGAATTCGACAGTTAGCGGAAACGCCCAC	This work
gfp-18-F	CTAACTGTCGAATTCAGGAGGCGAATTCATGAGTAAAGGAGAAGAAC	This work
P20-F	TATAGGGCGAATTG**GAGCTC**CAGGACGAACCTAGCTGAAC	This work
P20-R	ATGAATTCGCCTCCTGAATTCGAATTCAGAGGTATCCTGATCCATTAG	This work
gfp-20-F	GATACCTCTGAATTCAGGAGGCGAATTCATGAGTAAAGGAGAAGAAC	This work
P23-F	TATAGGGCGAATTG**GAGCTC**GGTACCCGTGGAAAGCTGGCTGAAAG	This work
P23-R	ATGAATTCGCCTCCTGAATTCGTATCACAGGGAGACATAGG	This work
gfp-P23-F	CTGTGATACGAATTCAGGAGGCGAATTCATGAGTAAAGGAGAAGAAC	This work
P25-F	TATAGGGCGAATTG**GAGCTC**GGTACC GCGCAGAGGGTCCTCGACAG	This work
P25-R	ATGAATTCGCCTCCTGAATTCGAGTCGGTTGTGTGTCTTG	This work
gfp-P25-F	AACCGACTCGAATTCAGGAGGCGAATTCATGAGTAAAGGAGAAGAAC	This work
P29-F	TATAGGGCGAATTG**GAGCTC**GGTACC GCACGGTTAGCCTGTTCG	This work
P29-R	ATGAATTCGCCTCCT GAATTCGTACATTCCAGACAATCTGC	This work
gfp-P29-F	GGAATGTACGAATTCAGGAGGCGAATTCATGAGTAAAGGAGAAGAAC	This work
gfp-R	CCGCCACCGCGGTG**GAGCTC**CTATTTGTATAGTTCATCCATG	This work
gfp-*lac*-F	GCCGCCACCGCGGTG**GAGCTC**AGGAGGCGAATTCATGAGTAAAGGAGAAGAAC	This work
gfp-*lac*-R	TATAGGGCGAATTG**GAGCTC**CTATTTGTATAGTTCATCCATG	This work
phaC1-PUF	CGC**GGATCC**GCCGAAGACGGCATCAAG	This work
phaC1-PUR	GAATTCTACATGACTAGGTACCGAGTCGCGCACTGGCCGTGTTC	This work
phaC1-PDF	GGTACCTAGTCATGTAGAATTCAGGACATTGGAGCGTCGTAG	This work
phaC1-PDR	CGC**GGATCC**AGGTCAGGGCATTGACTTTAG	This work
phaZ-UF	CAGGTCGACTCTAGA**GGATCC**TTCCTCGACGGCTTGCACGTAAC	This work
phaZ-UR	GCCGCAACCATTCGTGGTTCGCCAGCATTGAC	This work
phaZ-DF	CAATGCTGGCGAACCACGAATGGTTGCGGCATGGG	This work
phaZ-DR	GAGCTCGGTACCCGG**GGATCC**ACCGGCAGCAAAGACGTGAAC	This work

gfp-QF	TGCACTACTGGAAAACTACC	This work
gfp-QR	ACTCGATGCGATTAACAAGG	This work
phaC1-QF	GGCACTCAAACAACCCTTCC	This work
phaC1-QR	TCACCGTCTTCGGGCTTTAG	This work
phaZ-QF	TGGTGCCACTGCTGATCTTC	This work
phaZ-QR	TGGCCGTAGTCGAGGTAATC	This work
phaC2-QF	GCACCTCAATCCCTTCTATC	This work
phaC2-QR	TGTTGAACAGCTCCTTTACC	This work
16S-QF	TCACGATTACTAGCGATTCC	This work
16S-QR	AACCCTTGTCCTTAGTTACC	This work

P4:

GAGGTTTCCTGTGGTGAGCACGGAGTGCGGGTGATTTGTGCGTTTTACTTAACACATGAACCTGCACA
AAGGAATAGCCGGGCCTGATTTTCAAACATATTCGCGCGCTCTTCCCCCTGTGCAACGCCTCGAACGAT
CGTCAATTAAAATTTACAACGCAAGCGTACGCCAGTGCCGGATCGAATCACCATCAGCCGCCTCGATAG
TTACCATGCACACCATTCACTTCTGTGATTGCCAGAGCGCGAGGAATATCGACACATCGGGCAGTCGCC
CTCATCGCACAGAAGGAAACCGATCATGAGCATCTACGCCGCACTCGCCAGCTTCGCCGCCGCCTCCG
CCTTCCAGACCGCTGCACCGGTTCGTCCCGAGCAGGCCGCAGCCGCTGCTCGCGACACTCGTCCAAG
CTGACACGCCCAC

Start	End	Score	Predicted promoter sequences:
173	218	0.56	TGCCGGATCGAATCACCATCAGCCGCCTCGATAGTTACCATGCACACCAT

P6:

GGCTGAAGCGGCCTCCTGGGACTGCTGAAGAGGACGGATCGAGCTCGACCCGCGAGGGCCGCCAAG
CATACGCGTGCCGAACGCTCAGCGCGACGCCTTGCGAAACACCCGCCGCCAGGCTTGACACTCAGCC
AGAAGCACCGAGATAGAGCGCCCCGATCAAGTATTGAGGGGATCGATGTTCACTATCAGGTCAGTCA
AGTTCTTATCTTCGCTCATGTCCTCAAGAATTGGCTCCGAAGCCACTTATTACTCCCCAAC

Start	End	Score	Predicted promoter sequences:
178	223	0.82	GATGTTCACTATCAGGTCAGTCAAGTTCTTATCTTCGCTCATGTCCTCAA

P9:

CCGCTGCGCCACGGCCGCTCCGGCCGTGGCCTGCTGCGCCTTTCACAAGACCTTGAGCGCTCTATTTC
GGGCGTTTCAGCAAGCATCAGCGGCATCGATGTTCACCATCATGCCAATGAAGTTCTCTGAAAAAAT
CCAGTGCGTAACATCTGCTC CATACCCAACCACAACCCCGCT

Start	End	Score	Predicted promoter sequences:
115	160	0.65	AATGAAGTTCTCTGAAAAAATCCAGTGCGTAACATCTGCTCCATACCCA

P16:

ATCCGAGGGATACGGGCCTTGTCAGCACGGTGTTGCTAATGAGAGCCTTTGCCCGGGCAATAGTACG
GGCAGTGTGTAGCGGATTGAAAGACGCTGAATCACTGACAGGCATGAAGACTATCGATAGAGTCTGA
TAGTGTCGCCGCCGCACAGCGGATAGAGTCCACAGTCATTGAAGTGTTAATCCGCGATCAAGCTC

Start	End	Score	Predicted promoter sequences:
96	141	0.89	GAATCACTGACAGGCATGAAGACTATCGATAGAGTCTGATAGTGTCGCCG
129	174	0.86	GTCTGATAGTGTCGCCGCCGCACAGCGGATAGAGTCCACAGTCATTGAAG

P17:

GCCAGATCGACTGAAGGCACAAGGTCTTCAGGCCAAAGTGTTCCGGCGGCGGGTCGAATAGGGCCCG
TCTCTTACACAAGGCAAC

Start	End	Score	Predicted promoter sequences:
5	50	0.82	GATCGACTGAAGGCACAAGGTCTTCAGGCCAAAGTGTTCCGGCGGCGGGT

P18:

AGCTTCAAGCGGCAAGCCACTAGCTTCAAGTAAGGGCAGAAGCGATACGGAGTGGTTGATAGGCTTC
GGGCGGGCTTGCAACTTGCGGCTTGAAGCTTGCCGCTGCTCCTAGTATGTGATTTCTAATTTTTCTCCC
TAGGGTGTACCCAAGCCTGCATTCCGTGGGCGTTTCCGCTAACTGTC

Start	End	Score	Predicted promoter sequences：
95	140	0.85	GCTTGCCGCTGCTCCTAGTATGTGATTTCTAATTTTTCTCCCTAGGGTGT

P20:

CAGGACGAACCTAGCTGAACTCGGTCTGTCTGAGTTAACAATTGTTAAGACTCAGCCGTGACAGTTAC
GCGCGCCCGGCGTCTAATGGATCAGGATACCTCT

Start	End	Score	Predicted promoter sequences：
52	97	0.92	TCAGCCGTGACAGTTACGCGCGCCCGGCGTCTAATGGATCAGGATACCTC

P23:

TAGTCGTCATATGGCGGCAACGCCGTGGAAAGCTGGCTGAAAGCTTTCTACGGCATGCTGTACCCAC
ACGGCGCACCTGCCTGCAGGGGCTACCCGCCCAGCGCCGTGCCATCTCCTCGTGACCTTAGGGTCTGC
GCCAGCCTACCCGGCCGGTGCTTCGAGCGGGGCCAAACGCCCCGCCCATTTCCCTCCCCATTCTGATT
TCATCTCAACTGGCCATCATTGCGTAACGAAAAGTTCGGACTATGGTGTCAGGACGGTCGCCTTGCCG
CGCCCGCCAGAGCTGCAGTCCTGAGGCTTATCAGGTGCTCGACAACGCTTACTACACGGATGTGCGTT
GACGAATCCTGACTGTTGACTCAAGTCAGGATGTTCTGCTCGCCAAACGCGCTCAATACCTATGTCTCC
CTGTGATAC

Start	End	Score	Predicted promoter sequences：
216	261	0.57	GCCATCATTGCGTAACGAAAAGTTCGGACTATGGTGTCAGGACGGTCGCC
331	376	0.55	ATGTGCGTTGACGAATCCTGACTGTTGACTCAAGTCAGGATGTTCTGCTC

P25:

GCGCAGAGGGTCCTCGACAGACAGGCTTGCTTCAAAAAGAGAAACATTATCATCTAGAGATGCATTT
GAAGCAATGGACAAAACACCGCAGCAGAGCCCGTCGATCTATTGAGCCAGGTCAAAAAGTGGATTGT
TGCTTGGCTTAGCATTGATTCCAGCCAAGACACACAACCGACTC

Start	End	Score	Predicted promoter sequences：
19	64	0.51	AGACAGGCTTGCTTCAAAAAGAGAAACATTATCATCTAGAGATGCATTTG

P29:

GCACGGTTAGCCTGTTCGAACCAGAAGGGCTCCCGCGGGAGCCCTTTTTTGTGCCCGTCCGCCAGGTT
TGAGGGGGATTCTCTCGCTGCGCCCGCGGGCGCGGGATGTAGCGTCAGCGGGGACGTTCTCGCCTCG
TCTTGCGTAGCTGCCAGGCACTTTGCTTGTCCCTTTCACGGATGACCTTCGCTCGACCTCTTTTCGTCT
TGGGACATGCCCTGTACGGCAGTACCTAAAGATCATGCAAATTGCCCGAGAGATTTTTTCAGACTCGG
CGAAATGCAATTAAAAGCCTTCTTTGATTGATTTCTAAGTTATTGTTTTTAAAGAATTTATAAGTTTTT
TAAAAACTGGCACAGCGCTTGCGATAGTACTGGTGAACCTGCAGCCAGGAGTTGGTTTGCAGATTGT

CTGGAATGTAC

Start	End	Score	Predicted promoter sequences：
255	300	1.00	AGATTTTTTCAGACTCGGCGAAATGCAATTAAAAGCCTTCTTTGATTGAT
295	340	0.88	TTTGATTGATTTCTAAGTTATTGTTTTTAAAGAATTTATAAGTTTTTTAA
313	358	1.00	TATTGTTTTTAAAGAATTTATAAGTTTTTTAAAAACTGGCACAGCGCTTG
335	380	1.00	AGTTTTTTAAAAACTGGCACAGCGCTTGCGATAGTACTGGTGAACCTGCA

Fig. S1 Cloned promoter sequences and predicted promoter sequences. Each cloned promoter sequences were submitted into the web-based promoter prediction platform (Neural Network Promoter Prediction, http://www.fruitfly.org/seq_tools/promoter.html) and then the predicted promoter sequences were shown and scored. The transcription start was shown in larger font.

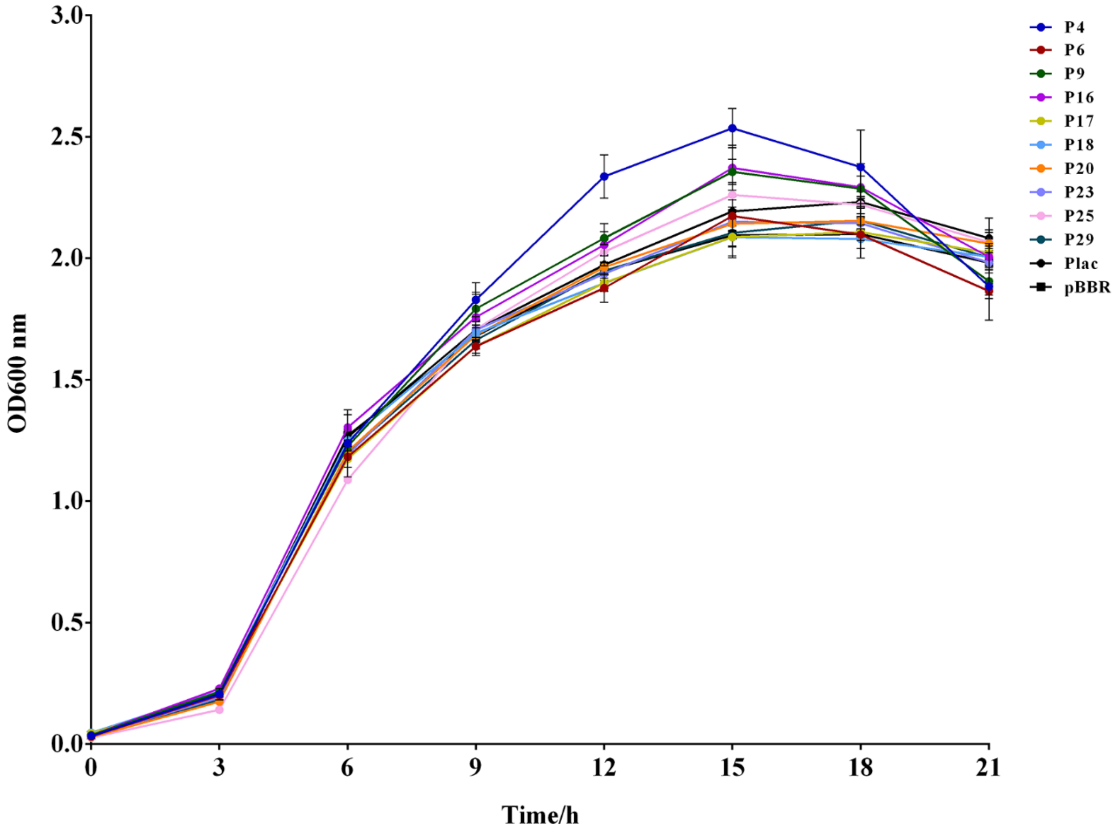

Fig. S2 The growth curves of all the constructed strains for promoter characterization. Data represent the mean values ± standard deviations of triplicate measurements from three independent experiments.

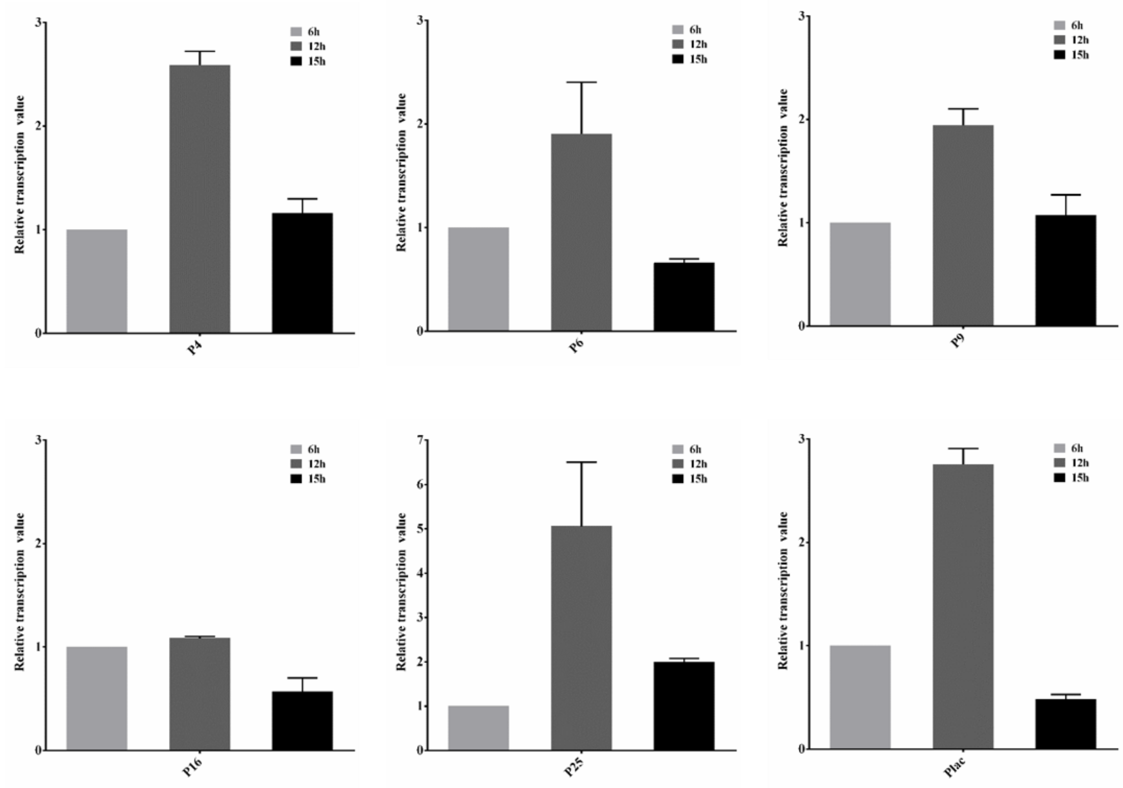

Fig. S3 Transcriptional levels of the five strong promoters and *lac* promoter in different growth phases. The relative transcriptional value of each promoter in early log phase was set as 1. Data represent the mean values ± standard deviations of triplicate measurements from three independent experiments.

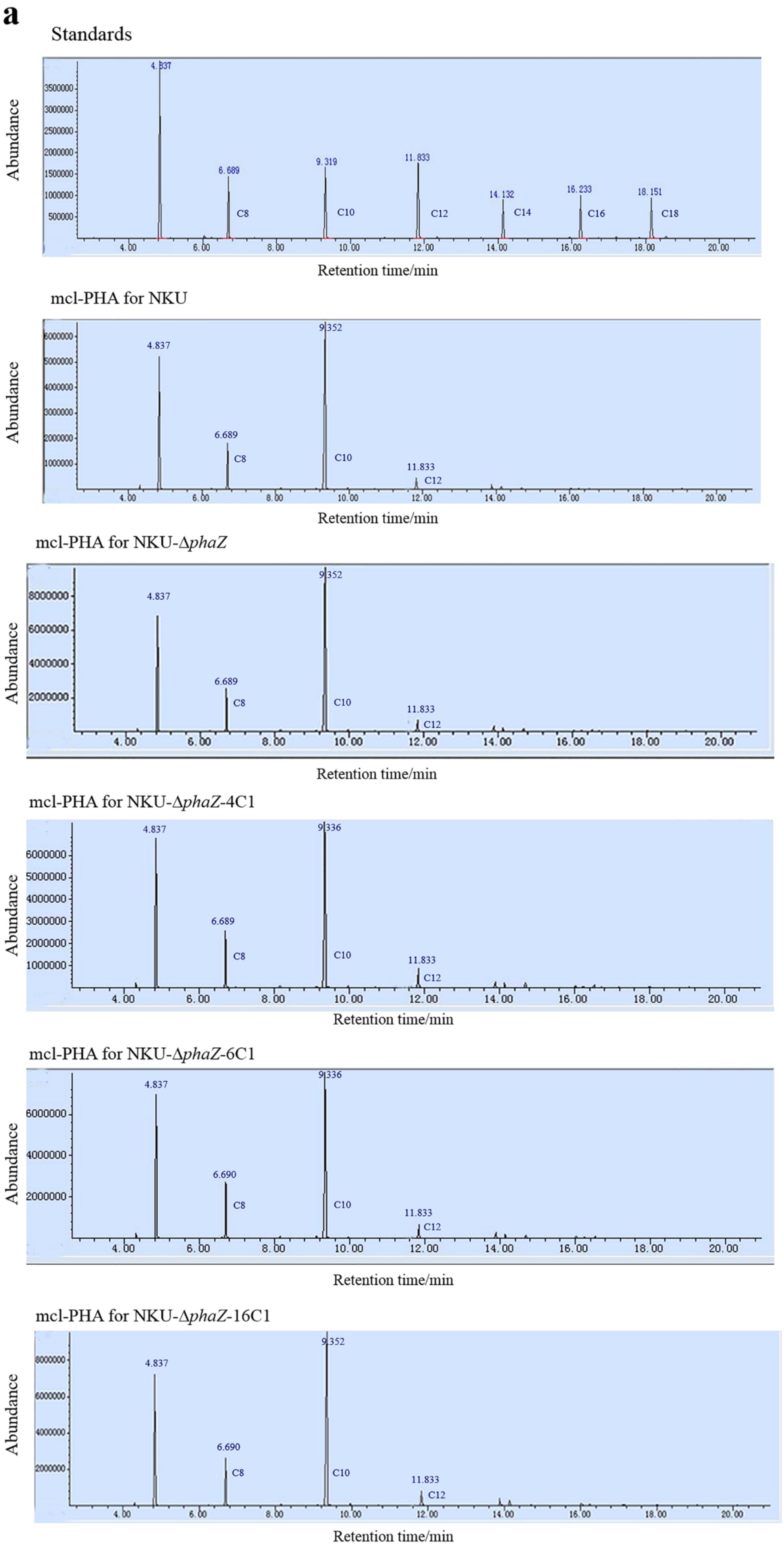

a
Standards
Abundance
Retention time/min
4.837
6.689
C8
9.319
C10
11.833
C12
14.132
C14
16.233
C16
18.151
C18
mcl-PHA for NKU
Abundance
Retention time/min
4.837
6.689
C8
9.352
C10
11.833
C12
mcl-PHA for NKU-Δ*phaZ*
Abundance
Retention time/min
4.837
6.689
C8
9.352
C10
11.833
C12
mcl-PHA for NKU-Δ*phaZ*-4C1
Abundance
Retention time/min
4.837
6.689
C8
9.336
C10
11.833
C12
mcl-PHA for NKU-Δ*phaZ*-6C1
Abundance
Retention time/min
4.837
6.690
C8
9.336
C10
11.833
C12
mcl-PHA for NKU-Δ*phaZ*-16C1
Abundance
Retention time/min
4.837
6.690
C8
9.352
C10
11.833
C12

b

C8-MS

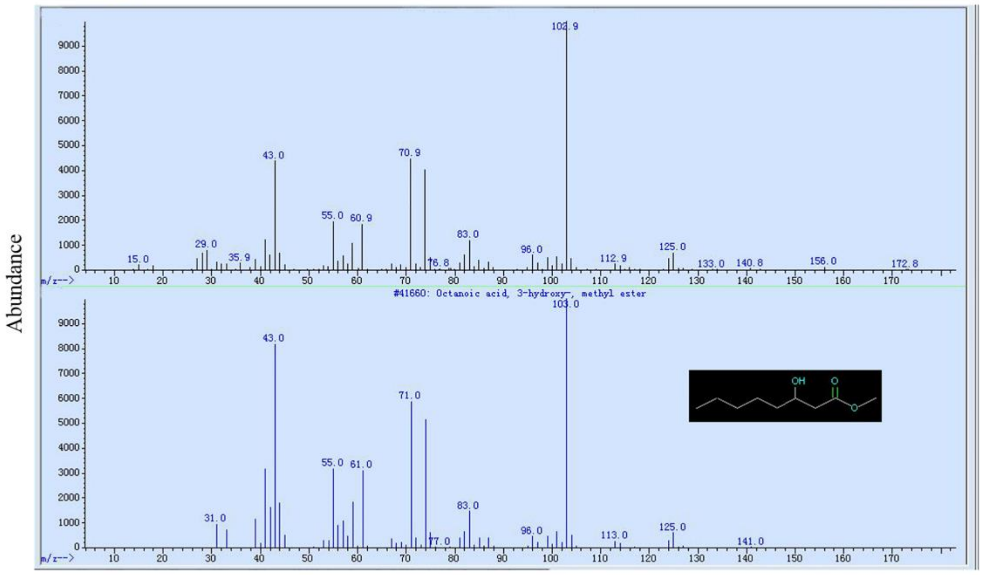

C10-MS

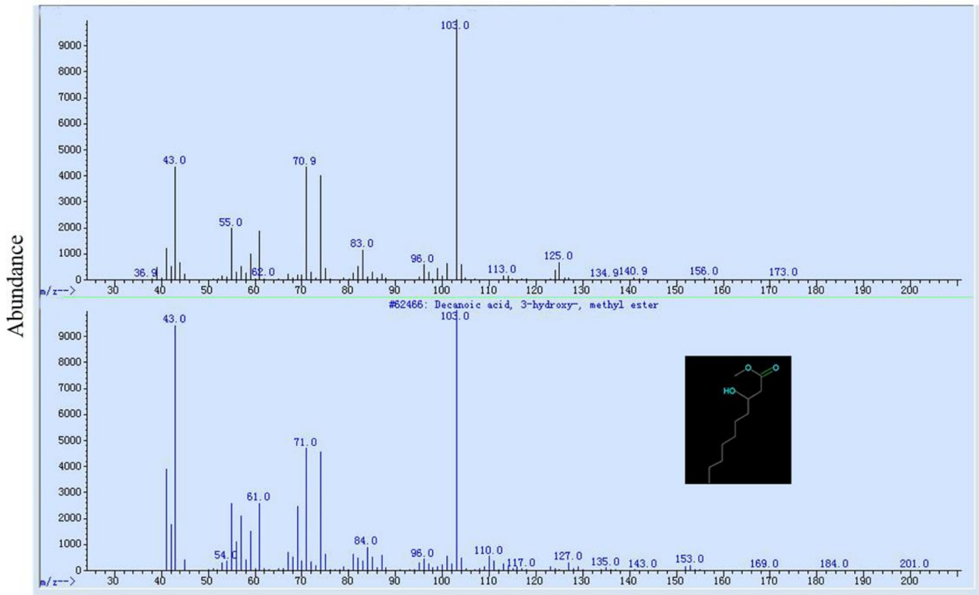

C12-MS

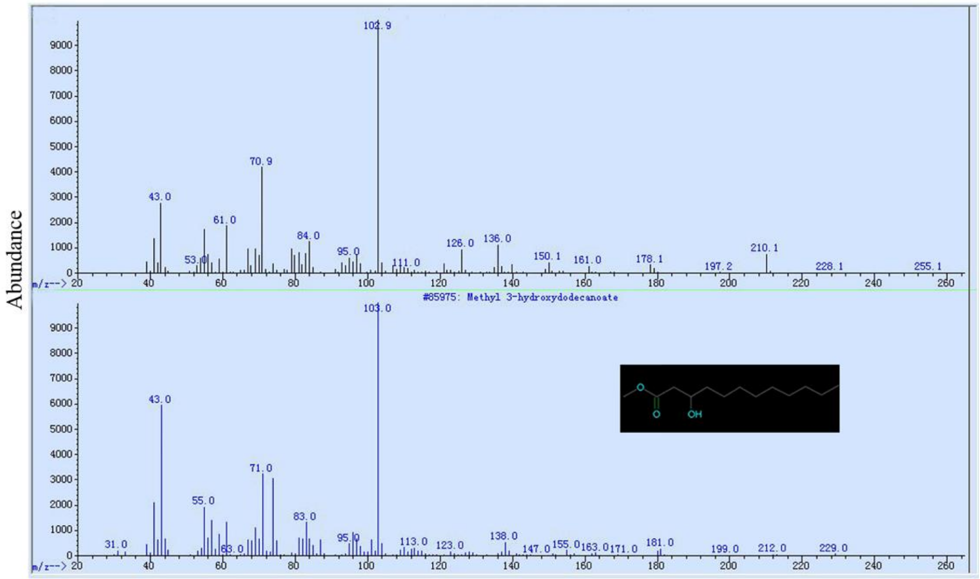

Fig. S4 GC-MS analysis for the monomer compositions of mcl-PHAs synthesized by *P. mendocina* NKU and its mutants. (a) The chromatogram for the various standards of methyl-3-hydroxy fatty acids and for the various monomers generated by the acidolysis of mcl-PHA. (b) The mass spectra of the various monomers generated by the acidolysis of mcl-PHA. Prior to GC-MS analysis, mcl-PHA sample was pretreated with H_2SO_4 and CH_3OH at 100°C for the acidolysis and methyl esterification. The peak in 4.387 min was methyl benzoate which was acted as the internal reference.

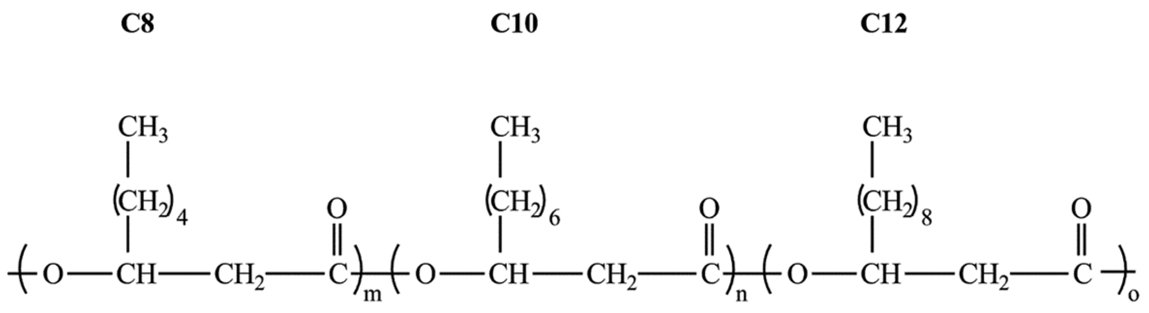

Fig. S5 The molecular structure of mcl-PHA produced by *P. mendocina* NK-01

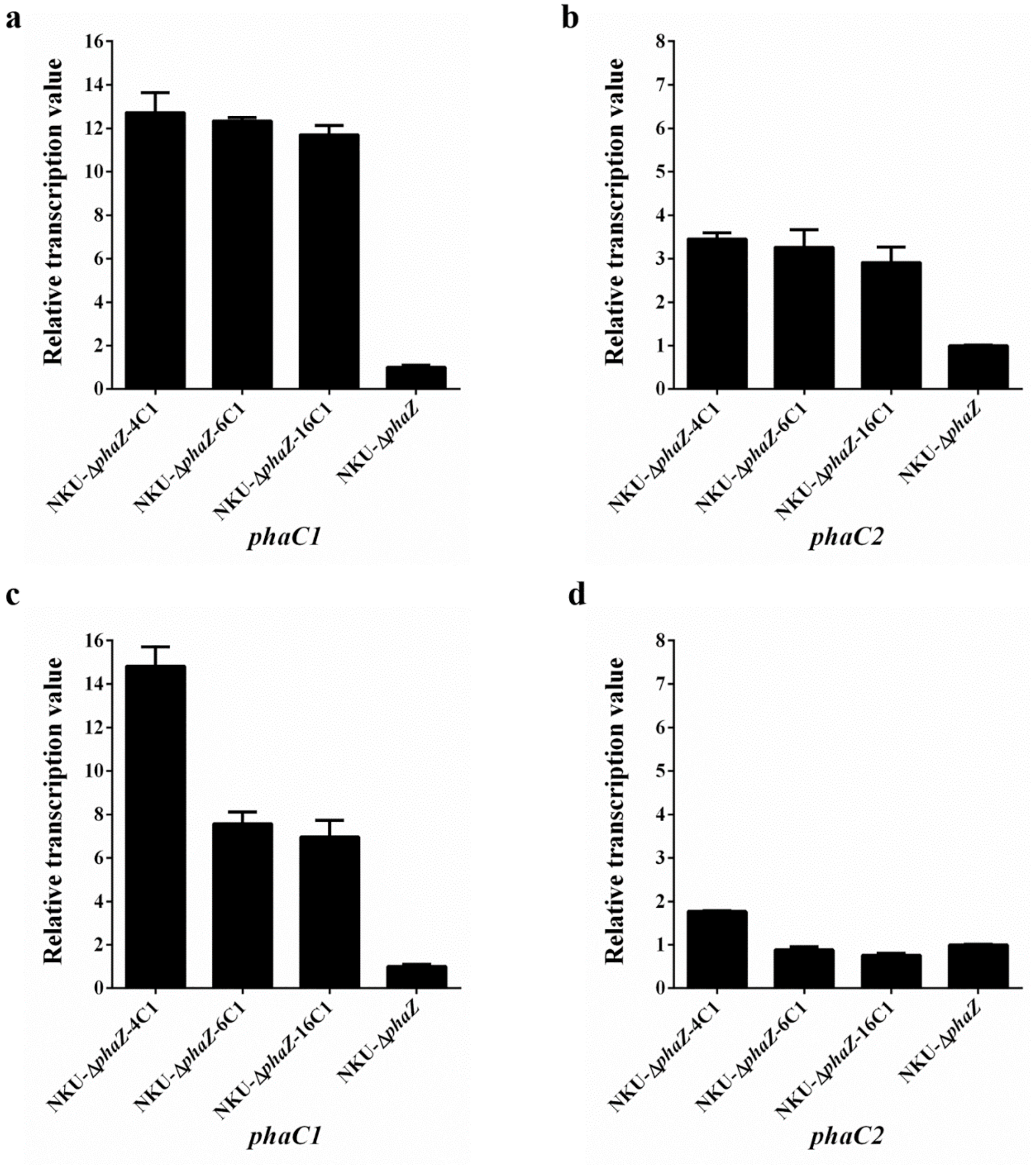

Fig. S6 Transcriptional levels of *phaC1* and *phaC2* for the different mutant strains at 12 h and 24 h. (a) Transcriptional levels of *phaC1* in 12 h. (b) Transcriptional levels of *phaC2* in 12 h. (c) Transcriptional levels of *phaC1* in 24 h. (d) Transcriptional levels of *phaC2* in 24 h. The transcriptional level for strain NKU-Δ*phaZ* was set as 1. Data represent the mean values ± standard deviations of triplicate measurements from three independent experiments.
